# Early access schemes for innovative health technologies: the views of international stakeholders

**DOI:** 10.1017/S0266462323000429

**Published:** 2023-07-06

**Authors:** Caroline Farmer, Brian O’Toole, Maxwell S. Barnish, Laura A. Trigg, Samuel Hayward, Louise Crathorne, Zelie Kasten, John Spoors, G. J. Melendez-Torres

**Affiliations:** 1 Peninsula Technology Assessment Group (PenTAG), Department of Public Health and Sport Sciences, University of Exeter Medical School, Exeter, UK; 2 Medicines Value and Access Unit, NHS England, London, UK; 3 North Somerset Council

**Keywords:** health policy, technology assessment, biomedical, insurance, health, reimbursement, therapies, investigational

## Abstract

**Objectives:**

Early access schemes (EASs) are approaches used by payers to balance and facilitate earlier patient access to innovative health technologies while evidence generation is ongoing. Schemes require investment from payers and are associated with significant risk since not all technologies will be routinely reimbursed. The purpose of this study was to gain the perspectives of policy experts about the key challenges for EASs and potential solutions for their optimal design and implementation.

**Methods:**

Two virtual workshops were convened including (i) UK-based policy experts (England, Wales, and Scotland) and (ii) representatives from multiple healthcare systems (England, France, Sweden, Canada, Poland, and Norway). Participants were encouraged to share their experiences with EASs in their healthcare system and highlight key challenges for policy makers. Discussions were transcribed and analyzed using framework analysis.

**Results:**

Participants agreed that EASs have value when targeted toward innovative technologies with the potential for significant clinical benefit in an area of high unmet need. Participants discussed potential solutions to the challenges faced by payers implementing EASs, including defining eligibility criteria, supporting evidence generation, and approaches to reimbursement.

**Conclusions:**

Participants agreed that EASs are one possible solution for their healthcare systems and have the potential to deliver significant clinical value to patients. However, widespread adoption of EASs is limited due to concerns about the risks for patients and healthcare budgets, further solutions are needed to deliver EASs for targeted therapies.

## Introduction

Early access schemes (EASs) include a variety of strategies that may be used by healthcare systems to facilitate earlier access to innovative health technologies where these present challenges for evidence-based reimbursement. These technologies, including advanced therapy medicinal products (ATMPs), are typically associated with greater uncertainty as clinical evidence is immature and there may be uncertainty about the generalizability of trial populations. Complex technologies may also present a significant step-change in care that poses a challenge for implementation, including alterations to service configuration, diagnostic requirements, and treatment pathways. However, while the value of these technologies for payers is uncertain, they are often targeted at indications with serious disease burden and a significant unmet need.

EASs can use a variety of approaches to support technologies, such as market-specific advice to manufacturers, streamlining of licensing and regulatory pathways, support with evidence generation, and commercial solutions such as performance-based reimbursement. There is often significant support for EASs from patients, clinical communities, policy makers, and the manufacturers of innovative technologies. Several healthcare systems have recently implemented schemes (such as the innovative licensing and access pathway (ILAP) (1) in England and Wales, the *Autorisations temporaires d’utilisation* (ATU) scheme in France (2), and Project Orbis (3), which is a cross-national initiative involving the USA, Australia, Canada, Singapore, Switzerland, Brazil, England, and Wales). However, there is significant uncertainty amongst stakeholders about the implications of these schemes for patients and healthcare budgets, and it is likely that payers will adapt or create new schemes to meet the changing demands of growing innovation in health care. The aim of this research was to draw upon the expertise of policy makers from multiple healthcare systems to consider the role of EASs in promoting access to innovative pharmaceuticals. In particular, we were interested in understanding stakeholders’ perceptions of the key aims for developing EASs in their countries, and the way in which evidence generation and commercial strategy could be used to overcome challenges with implementation. Overall, we aimed to develop a set of guiding principles for the way in which EASs should be designed and implemented. The findings of the research have been shared with policy makers in England and participating markets to assist with developing their strategic thinking on EASs.

## Methods

Two 2-hr workshops were conducted with policy makers involved in the regulatory approval of medicines who possess experience or interest in early access to medicines schemes. One workshop was attended by representatives from the UK (England, Wales, and Scotland), and the other workshop was attended by representatives from across multiple healthcare systems (England, France, Sweden, Norway, Poland, and Canada). To overcome scheduling difficulties for the workshops, a separate 30-min interview was conducted with one stakeholder who was unable to attend the UK workshop.

### Participants and Recruitment

Purposive sampling was used to recruit participants for the two workshops, shown in Table 1. Participants were senior staff in agencies responsible for appraising the evidence base of health technologies, engaged in health policy, or involved with commercial negotiation for health technologies. Direct experience of an active EAS was not required for participation, given that not all countries would necessarily have active schemes at the time. It was anticipated that participants without direct experience would nevertheless be cognizant of the key issues in their healthcare systems that EASs may address, and may have been involved in high-level policy discussions about the design and potential implementation of a EAS in their country. In addition, two academic stakeholders attended from the University of York and the London School of Hygiene and Tropical Medicine, respectively, both experts in the health economic evaluation of health technologies. In total, thirteen participants attended the UK workshop and ten participants attended the international workshop. In advance of the workshop, participants were sent a slide deck containing the full topic guide for the workshop and some introductory slides about the topic area and the key issues that had prompted the research. Attendees of the international workshop also received a summary of the initial findings from the UK workshop.

**Table 1.** Organizations represented at each of the workshops

UK-based workshop	International workshop
National Institute for Health and Care Excellence (NICE) (*N* = 2)	National Institute for Health and Care Excellence (NICE) (*N* = 1)
NHS England (*N* = 3)	NHS England (*N* = 3)
Scottish Medicines Consortium (SMC) (*N* = 1)	*Haute Autorité de Santé* (HAS) (*N* = 1)
All Wales Medicines Strategy Group (AWMSG) (*N* = 1)	The Swedish Dental and Pharmaceutical Benefits Agency (*N* = 1)
The Medicines and Healthcare products Regulatory Agency (MHRA) (*N* = 1)	Norwegian Hospital Procurement Trust *N* = 2)
All Wales Therapeutics and Toxicology Centre (AWTTC) (*N* = 1)	Agency for health technology assessment in Poland/*Agencja Oceny Technologii Medycznych i Taryfikacji* (AHTAPol/AOTMiT) (*N* = 2)
Department for Health and Social Care (DHSC) (*N* = 2)	Canadian Agency for Drugs and Technologies in Health (CADTH) (*N* = 1)
University of York (*N* = 1)	
London School of Hygiene and Tropical Medicine (*N* = 1)	

### Online Survey

Prior to the workshop, stakeholders were invited to complete an optional survey used to inform the workshop schedule. The survey took 5–10 min to complete and contained a mix of closed (yes/no and multiple choice) and open questions (free-text) about stakeholder experiences of early access to medicines schemes and their key challenges. The results of the survey were not analyzed for inclusion in the final results.

### Data Collection

Workshops each lasted 2 hr and the individual interview with the UK stakeholder lasted 30 min. The schedules were semi-structured around key topic areas for early access to medicines policy: general principles for the design and implementation of EASs, evidence generation and appraisal, and commercial strategy. The schedule was adapted for each session, both to target the expertise of the particular group and to reflect the researchers’ evolving understanding of the key issues for early access policies through reflexive practice (4). The topic guides used to guide discussion in each workshop are provided in the Supplementary Material.

The workshops and interview were conducted online using Microsoft Teams. Members of the academic team chaired the discussions (first and second authors), asking questions and prompts as needed. Participants were invited to use the chat function during the workshops, and these contributions were included in the analysis (labeled as text). A recording of the sessions was made for the purposes of transcription, after which it was deleted.

### Analysis

Transcripts were analyzed using framework analysis (5). This approach codes participants’ responses using methods consistent with thematic analysis (6) though codes are integrated within a “frame” of the key areas of interest as set by the research team *a priori.* For this study, the frame was informed by previous research to evaluate the use of performance-based managed access agreements (MAA) in England and Wales (7) and amended flexibly during the analysis. The final frame is outlined below in Table 2.

**Table 2.** Themes identified in the analysis

Framework	Themes	Theme description
Aims of EASs	Access to promising treatments	Delivering (early) access to promising technologies was considered to be a valuable goal of EASs
	Facilitating decision making	EASs could be used to reduce uncertainty in the evidence base and aid decision making. However, the potential for withdrawing treatment at the end of the EAS complicates decision making
	Streamlining regulatory timelines	EASs may be used by payers to align timelines for the appraisal of technologies for the same indication together, and to reduce the time period between product licensing and patient access
	Supporting innovation	Domestic stakeholders discussed EASs could be used to support the life sciences industry in developing innovative medicines
Eligibility	Uncertainty in the evidence base	Where uncertainties in the evidence base prevent an understanding of the clinical and cost-effectiveness of a technology sufficient for decision making
	Feasible	Where the EAS is likely to enable a decision on routine commissioning at the end of the agreed time period
	Necessary/justified	EASs should be used secondary to routine commissioning with less complex access schemes, such as the use of confidential discounts, and where the risks and the resource required to conduct the scheme is justifiable to the expected benefits
	Innovative	EASs should place focus on innovative technologies that present challenges for routine pathways to access and plausibly offer significant benefits in an area of high unmet
Context	Broader health policy	There was uncertainty from stakeholders about how EASs fit alongside existing commercial agreements with industry. Further discussions are needed to approach this strategically and consider, for example, how the relative risks and benefits shared by payers and industry fits into broader strategy. EASs may also impact policies on indication-based pricing, and stakeholders were unsure about how to navigate this
	Role of reimbursement agencies in EA	Reimbursement agencies have a key role to play in designing and coordinating EASs. Stakeholders expressed concerns about the extent to which the payers would be expected to take on a research role and have responsibility for evidence generation
	Role of patients in EA	Further thinking is needed about the role of patients in EASs. There was support for engagement with patients about the way exit strategies requiring the withdrawal of care should be implemented
	Influence of the healthcare system	It was noted that regionalized healthcare systems present particular challenges for EASs, including equity of access and coordinating pricing strategy
Implementation	Horizon scanning	Increased horizon scanning would increase the efficiency and quality of EASs
	Planning	Companies should submit an application for EA outlining the need and proposed approach for the EAS. An early multi-stakeholder meeting(s) should be conducted to determine eligibility and create an action plan for the scheme
	Length of interim access period	Shorter or capped time periods for EASs were considered preferable, where possible. Stakeholders expressed concerns that timelines may slip for many reasons, and that this will impact on the balance of risks and benefits of the scheme to payers and industry
	Managing resource	Stakeholders anticipate that EASs will require significant resource from teams in reimbursement agencies, in addition to resource from health services engaged in data collection
	Sharing resource burden	A decision is needed on the way the required resource will be shared between payers and industry. On the whole, stakeholders considered that resource should follow the expected balance of risks and benefits between industry and payers, with the majority of resource funded by industry. It was also noted that sharing resource needs across payer organizations, including across regions and devolved nations, would increase efficiency
	Contracts	Stakeholders discussed the need for contracts to incentivize companies to engage. It was noted that contracts need to be clear with respect to exit strategies, changes in the length of the time period, and responsibilities between payers and industry
Evidence generation	Data collection plans	Data collection plans should be feasible, clearly linked to the requirements for decision making, and should adopt gold standards for research. Stakeholder engagement should be used to identify appropriate outcomes, and plans should consider how the analysis will consider emerging comparators. Payers may wish to augment data collection for other purposes, and this should be factored in when considering the relative benefits and responsibilities for data collection
	Types of evidence	RWE, PROs, and financial data may be useful to collect during EASs, though all are associated with known limitations that may limit their usefulness for informing decision making. There is a need to improve methods in these areas. It may not be justified to agree an EAS if trial evidence is forthcoming
	How to collect data	Innovative solutions to collecting and monitoring data collection may be needed to ensure quality. These solutions may require significant resource, and it is currently unclear who will fund this and take responsibility for data collection
	Evidence appraisal	The timing of evidence appraisal may need to be determined on a case-by-case basis, according to the data collection plan. There was an appetite for a lighter evidence appraisal to inform eligibility for EA, followed by rolling review through the EAS, and a full appraisal at the end of the scheme to decide on routine commissioning
	Accounting for uncertainty	There was support for the recommendations in the NICE methods review in relation to methods for interpreting uncertainty. For example, the characterization of uncertainty, greater exploration of the impacts of uncertainty, and the potential role of further evidence generation in resolving uncertainty. It was recommended that these methods are trialed before wider rollout
Commercial strategy	Reimbursement	There is a need to develop flexible reimbursement solutions for use within EASs. At present, options such as outcomes-based pricing and “subscription” plans face a number of barriers to implementation and the broader impacts of these schemes require thought. These could be applied universally or on a case-by-case basis. Decisions about reimbursement should be informed by the balance of benefits and risks for a specific technology, and considerations of how reimbursement affects the healthcare setting more broadly
	Pricing strategy	The simplest route to reimbursement is preferred, such as simple discounts within a patient access scheme, and companies should submit a rationale if they consider alternative approaches are required

EA, early access; EAS, early access scheme; PRO, patient-reported outcome; RWE, real-world evidence.

A matrix was developed for each research question with evidence from each session coded and allocated across the developing themes. Anonymized quotes were used to exemplify key themes, where informative. Several iterations of the themes were built, with each iteration working to further refine the themes. The analysis was conducted by a single academic researcher, and codes at each iteration were checked for completeness and validity by a second academic researcher. Code summaries were shared across the research team, and discussion within the team informed further development of the themes.

### Ethical Approval

The study gained appropriate ethical approval from the lead author’s host institution.

## Results

An overview of the results is provided in Table 2.

All countries represented in the workshops had current policies in place to facilitate patient access to health technologies outside of routine commissioning pathways, however, participants considered that their existing schemes did not fully address the challenges with innovative technologies. Some countries were further ahead in implementing new policies to address the challenges than others, though most participants stated that there were delays in policy making due to concerns about how the increased risk of these health technologies could be managed, particularly with regard to commercial strategy. However, all participants acknowledged that earlier patient access to pharmaceuticals was a current topic for discussion amongst regulators in their countries, and there was collective interest in considering ways in which these schemes could be utilized.

### Aims and Eligibility: Key Aims for EASs

Participants considered that the design of EASs will depend on the key barriers to reimbursement for the technology. There may be barriers specific to each healthcare system, though common barriers that would be feasible targets for EASs were discussed by participants, and included: (i) accelerating timescales to commissioning by streamlining appraisal processes; (ii) increasing the number of appraisals considering multiple technologies for the same indication; and (iii) facilitating evidence generation to resolve uncertainties in the evidence base.

Early access policies have the potential to accelerate timelines by facilitating greater collaboration between licensing and regulatory bodies. Participants considered that conceptually licensing bodies were the “fast track” compared to “slow track” decisions by payers and that by finding efficiencies in the process and working closely together this perception could be challenged. In France, companies now submit a single dossier containing evidence to be assessed simultaneously by payer and licensing teams, and payers aim to deliver a recommendation on access within three months of the product license (2). Some efficiencies can be made through advanced planning, enhanced horizon scanning, and earlier consideration of evidence-generation challenges. However, participants flagged that timelines were often driven by global regulatory processes and that manufacturers are often unable to submit evidence or value propositions independent of this.

EASs may also be used to align timelines so that multiple technologies for the same indication in specific therapy areas can be appraised at the same time (called a multiple technology appraisal (MTA) in the UK). Whilst scheduling appraisals simultaneously can bring efficiencies in terms of evaluation and commercial negotiation, it is highly unlikely that individual product regulatory timelines will synchronize and therefore health systems face a dilemma of potentially delaying patient access to achieve efficiency – this is a scenario with greater consequences in therapy areas with a high unmet need.

Supporting evidence generation was considered to be a valuable aim of EASs, particularly when evidential uncertainties related to the generalizability of data to local settings, and where recruitment for clinical trials may be challenging. However, due to concerns about the resource implications of data collection for clinical staff and patients, participants considered that evidence-generation plans should closely align with the data that would facilitate both regulatory and payer decision making. Participants also noted that evidence generation should be feasible, taking into consideration the likely quality and follow-up of any additional data that would be generated.

**Figure 1.**
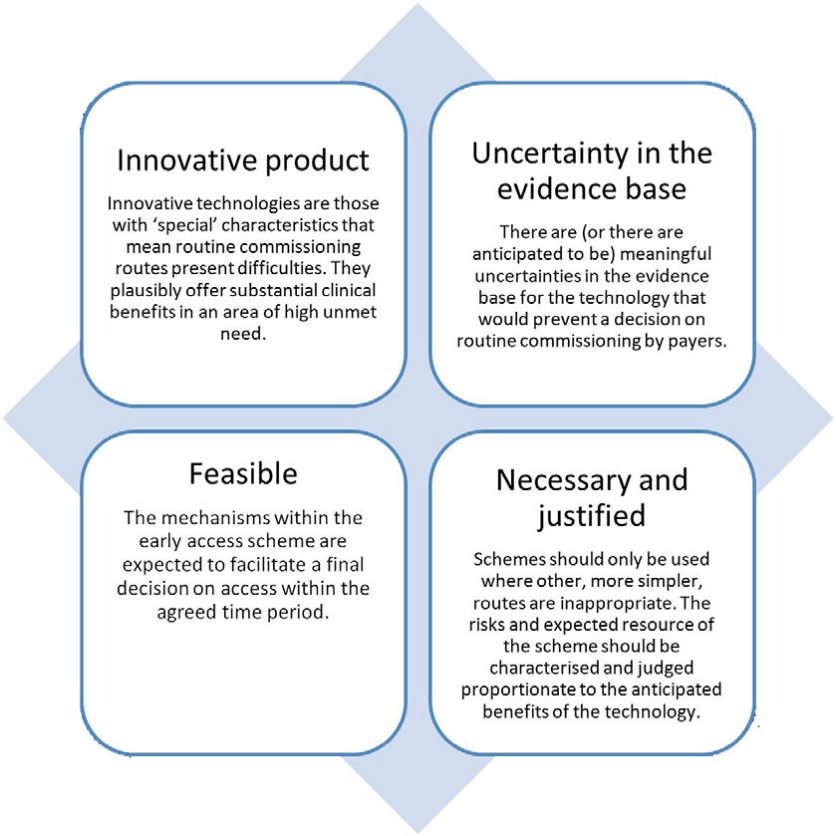
Proposed eligibility criteria for early access schemes.

Participants agreed on a number of general criteria that should be used to guide eligibility for EASs. These are depicted in Figure 1.

#### Proportionate Response

Participants discussed the risks of EASs and noted that schemes often require substantial resource to implement. It was therefore considered that the use of EASs should be targeted toward technologies where the resource needs were proportionate to the plausible benefit of the technology for patients. It was also considered that an early value assessment should determine the technology to be plausibly cost-effective and that any commercial strategy with industry should ensure that payers receive a fair balance of the benefits and risks from the EAS.

#### Assessing the Potential Value of the Technology

To determine whether EASs are a proportionate response, participants proposed that an early value assessment should be convened to determine whether the technology was likely to offer significant value in an area of unmet need. The assessment would require consideration of the level of unmet need and disease burden in the population and should be informed by broad engagement with stakeholders. Participants accepted that decisions on value will be complicated due to the immaturity of clinical effectiveness data, however, felt that early trial evidence may yet provide sufficient evidence of plausible value in areas where there is significant need. Early value assessments of technologies would be further supported by expanded use of horizon scanning, which would also provide an initial assessment of the potential for a new technology (this would also have benefits for implementation, as discussed later).

#### Feasibility Assessment

During the early value assessment, participants proposed that decisions on eligibility for an EAS should be informed by a feasibility assessment to assess whether the support provided by the scheme will feasibly lead to a final decision on whether the technology will be reimbursed. Several participants shared past experiences where a decision had not been possible, commonly because of failures in developing or implementing plans for evidence generation. In some instances, stakeholders considered that this eventuality was foreseeable. These instances place payers in a difficult situation, faced with the choice of increasing their share of the risk or withdrawing the technology from health services when patients have had prior access to a product.

### Context

Participants noted that the design of EASs will be affected by contextual factors. Key considerations include the nature of the healthcare system, how schemes in one stage of the appraisal timeline affect other stages, and how EASs fit within broader health policy. Contextual factors can alter the relative balance of risks and benefits shared between payers and industry, which may alter further if the context changes during the timeline of an EAS.

The relative balance of risks and benefits of EASs may vary according to the strategic importance of the healthcare system for the industry. Payers may wish to accept higher risks where this would support broader health strategy, such as policies toward innovation. Large markets or collaboratives between nations may also be able to negotiate more favorable terms. Participants noted that EASs in regionalized healthcare systems will need to incorporate additional complexity to ensure equity of access and coordination in pricing across the regions.

Payers should also determine whether the period of reimbursement within EASs is appropriate relative to the period of market exclusivity and in ensuring fair competition between comparators. Participants felt that the duration of reimbursement in EASs should be capped and that technologies should not be reimbursed through multiple schemes in the same market. It was noted that the period of reimbursement should also be considered with regard to the size of the incident population and future innovation in the area, so that the scheme does not facilitate unfair access to the market and disadvantage competitors.

Stakeholders considered that a holistic approach to designing EASs should be used to ensure that these fit alongside broader payer policy. A clear outline of the available routes to access will reduce the complexity of accessing schemes for industry, and payers should ensure that the relative benefits and risks of schemes agreed in negotiations take into consideration any broader agreements between payers and industry. Commercial agreements within EASs may be affected by broader pricing policy, for example, whether technologies are reimbursed at the same or different rate according to each indication (indication-based pricing), as well as broader collaborative agreements between healthcare systems and industry.

### Implementation

Participants considered that payers would benefit from expanding their use of horizon scanning to identify both technologies and indications that may be useful targets for an EAS. Early identification of indications would aid decision making in a subsequent early value assessment. Participants suggested that expanded horizon scanning may also be used to identify where real-world evidence sources for target indications are in need of development prior to evidence generation, thus allowing sufficient time for these to be prepared. It was also noted that investment in real-world evidence within healthcare systems could have broader value for healthcare systems, particularly where this involves the development of financial evidence systems.

It was also noted that EASs will require sophisticated contracts between payers and industry that clearly outline the responsibilities of each party and account for changes that may occur during the scheme. Participants noted that common changes that should be considered within contracts include changes in the product license, delays or failures in evidence generation, emergence of a new comparator, or parties failing to meet their obligations as specified (e.g., companies not submitting evidence generated during the scheme). In particular, participants emphasized the importance of clear exit criteria, and contracts may also wish to consider how prices will be determined when reimbursement will be based on performance (particularly where there is remaining uncertainty in the evidence at the end of the scheme). A lack of clarity about the responsibilities of payers and industry has at times caused breakdown in the relationship between payers and industry and increased the risk that decisions on routine commissioning are appealed. Participants expressed concerns that many payers will have limited legal resources compared to industry, and that this may expose payers to increased risk.

### Evidence Generation

Many stakeholders discussed experiences in which evidence generated during an EAS was limited in quality and/or did not fully resolve uncertainties in the evidence base. While participants considered that real-world evidence generated in the target population would be valuable, concerns about adequate control of confounding may be too great to fully inform decision making. In some cases, participants considered this issue may be foreseen by the implementation of a feasibility assessment that considered the likely quality of evidence to be collected, including the standard of available sources and the likely length of follow-up. Payers should have a clear strategy for how ongoing uncertainty in the evidence would be handled at reappraisal, such as whether they will permit an extended period of evidence generation. Methods for handling uncertainty, such as those recommended within the updated NICE health technology assessment (HTA) methods guide (8), may also be instrumental in this, though stakeholders noted that there was yet little experience with these methods and so to what extent they would be able to facilitate decision making. Participants also considered that stakeholders should be involved in specifying outcomes to be measured in evidence generation, to ensure that these best capture disease outcomes relevant to patients.

### Reimbursement

Participants felt that payers should reimburse technologies at prices proportionate to the evidence for their value. While this may only become sufficiently clear at the end of the scheme, it was considered unrealistic that technologies would uniformly be provided as free stock before this time. This would be particularly unfeasible for smaller manufacturers, extremely high-cost technologies (such as gene therapies), and technologies with very low incident populations. While some participants nevertheless considered it inappropriate that payers would reimburse technologies with unproven clinical and cost-effectiveness, most participants agreed that a price would need to be set for technologies during the EAS, though there was uncertainty about how this would be determined. In France, industry is permitted to set its own price during the reimbursement period, which would then be subject to a claw-back mechanism to recoup any funds paid over the final, “true” value determined at the end of the scheme. However, some participants were concerned that a claw-back mechanism would be challenging to implement in their healthcare systems. One stakeholder recounted an experience where evidence generated during a scheme suggested that reimbursement for the technology was too high, but uncertainty in cost-effectiveness estimates meant that they could not determine and justify a change of price.

Participants considered that EASs would benefit from a range of options for reimbursement, including innovative pricing solutions tied to the performance and uptake of the technology. However, as these are complex to implement and require payers to have sophisticated financial systems, participants agreed that simple pricing strategies such as the use of a flat discount rate (e.g., simple patient access schemes in the UK) tied to final cost-effectiveness estimates would be preferable.

## Discussion

EASs encompass a variety of approaches that can be used by payers alongside accelerated regulatory pathways to support access to innovative health technologies. However, implementation of such schemes has been limited due to uncertainty about the way these schemes can be designed and implemented to protect healthcare systems from substantial risk. This study draws upon the expertise of international stakeholders to identify key learnings and recommendations for designing and implementing EASs.

Overall, there was alignment between participants about the need and potential benefits of EASs, and criteria that could be used to determine eligibility. It was proposed that payers conduct an early value assessment, informed by consultation with clinicians and patients, to determine eligibility and feasibility for a technology, as well as the type of support that will be provided. In consideration of the significant risks and resource implications of EASs for payers, participants further agreed that the eligibility for EASs should be targeted proportionately to the expected clinical value of the technology for the healthcare system.

It is likely that significant changes in the infrastructure for health technology appraisal will be required for the effective rollout of EASs. This includes expanded horizon scanning processes, financial and methodological support for real-world evidence sources, a unified system for recording financial data across healthcare settings, developing legal expertise in contracting, and greater transparency and collaboration between regions and/or countries. Initiatives such as Project Orbis and ILAP draw upon the value of sharing data and resource across payers and provide solutions to the challenges faced by stakeholders with regionalized healthcare systems. It will be particularly useful for the UK to find further collaborative solutions to these issues following exit of the European Union and in the move toward greater regionalized health care through integrated care systems (9).

Payers should also consider strategies for pricing technologies reimbursed within EASs. There are a number of performance- or financial-based reimbursement models (10) that may be relevant for use in EASs. These include developing robust processes for implementing a claw-back mechanism, where payer recoup payments from manufacturers where technologies do not deliver the clinical benefits on which they were reimbursed, which may be informed by evaluations of the system introduced by France. Other solutions could include reimbursing the manufacturer at a low price calculated to cover essential costs, and for payers to reimburse the manufacturer according to the additional value of the technology as determined at commissioning. However, as noted by participants and by a recent review of these models (10), such models are complex to implement, particularly when payers are required to tie efficacy outcomes in an uncertain evidence base to payments. In the interim, payers are likely to continue a preference for simple reimbursement strategies such as the use of simple discounts, but where payers invest in financial systems and develop methods around performance-based reimbursement, this may open up new options that may suit early access.

A strength of this research is that the findings draw upon the considerable expertise of stakeholders with experience in developing and implementing health policy for appraising health technologies across various settings and payer contexts. However, not all payers were represented, and while those in attendance aimed to represent the experiences of their organizations, they will have been guided by their personal experiences. This means that not every encounter or barrier to EASs will be represented in the findings. Relatedly, within the time constraints of the project, there was a limit on the depth of discussion for each of the topics raised by stakeholders. While the research highlights important areas of agreement and gaps in understanding, further research is needed to fully explore the different mechanisms within EASs. Further research would also be needed to gather viewpoints from stakeholders in sufficient detail to allow for a comparison in views across countries, since attitudes to the role of EASs and their risks, and the ease with which EASs may fit into healthcare systems, is likely to vary.

## Conclusion

Demand for EASs is growing in response to accelerated regulatory processes for innovative technologies which meet an unmet need. EASs have potential to deliver significant benefits to patients and healthcare systems, but this needs to be balanced against the financial risk, infrastructure, and resource requirements to implement them. Enhanced horizon scanning and early stakeholder engagement would allow for earlier identification of promising technologies and would inform processes for evaluation. Increased capacity for real-world data collection will also be essential to the feasibility of schemes.

## Abbreviations

ATMPs: advanced therapy medicinal products
ATU: Autorisations temporaires d’utilisation
EAS: early access scheme
HTA: health technology assessment
ILAP: innovative licensing and access pathway
MTA: multiple technology appraisal

## Supplementary material

For supplementary material accompanying this paper visit https://doi.org/10.1017/S0266462323000429. click here to view supplementary material
