# Supplementary figures and images for: Early access schemes for innovative health technologies: the views of international stakeholders
Source: Int J Technol Assess Health Care. 2023 Jul 6;39(1):e45. doi: 10.1017/S0266462323000429 (PMC11570020; doi:10.1017/S0266462323000429)

Supplementary material

# ***Domestic workshop topic guide***


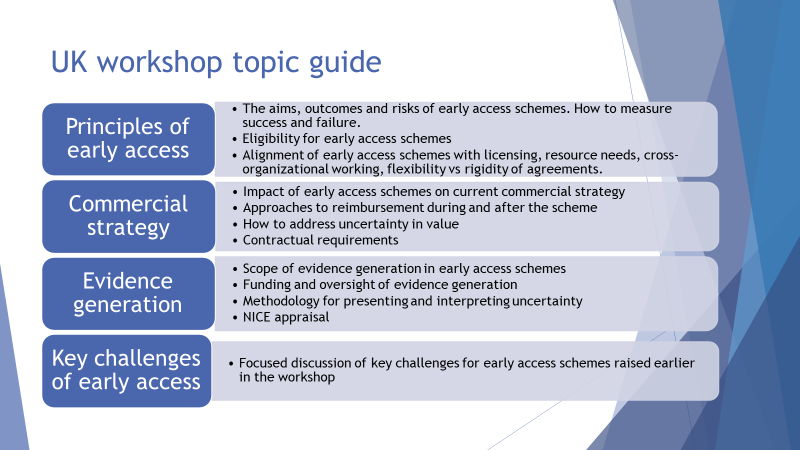


***International workshop topic guide***


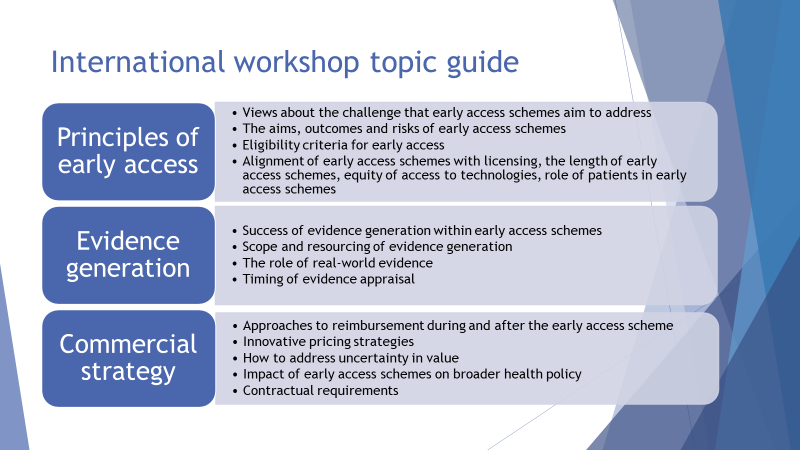

Supplement: Supplementary file 1 [file S0266462323000429sup001.docx]
